# Magnetically separable Fe_3_O_4_-SiO_2_/Pt catalyst and its application for uranium reduction

**DOI:** 10.1038/s41598-025-03867-y

**Published:** 2025-07-04

**Authors:** Kuntal Kumar Pal, Ramakrishna Reddy, Chanchal Ghosh, K. Ananthasivan, P. Velavendan, Ramesh L. Gardas, Sandip Dhara

**Affiliations:** 1 Reprocessing Material Development Section, Process Radiochemistry Reprocessing Research & Development Division, Reprocessing Group, Indira Gandhi Centre for Atomic Research, Kalpakkam, 603102 India; 2 Homi Bhabha National Institute, Anushaktinagar, Mumbai, 400094 India; 3 Minor Actinide Chemistry and Reconversion Section, Process Radiochemistry Reprocessing Research & Development Division, Reprocessing Group, Indira Gandhi Centre for Atomic Research, Kalpakkam, 603102 India; 4 Physical Metallurgy Division, Metallurgy & Materials Group, Indira Gandhi Centre for Atomic Research, Kalpakkam, 603102 India; 5 Reprocessing Group, Indira Gandhi Centre for Atomic Research, Kalpakkam, 603102 India; 6 Department of Chemistry, Indian Institute of Technology, Chennai, 600036 India; 7 Material Science Group, Indira Gandhi Centre for Atomic Research, Kalpakkam, 603102 India

**Keywords:** Magnetic nanoparticle, Catalysis, Uranium reduction, Fe_3_O_4_-SiO_2_/Pt, Hydrogenation, Chemistry, Energy science and technology, Materials science, Nanoscience and technology

## Abstract

Magnetically separable Fe_3_O_4_-SiO_2_/Pt catalysts with ~ 2% Pt loading have been developed for the generation of U(IV) in HNO_3_-N_2_H_4_ medium, used in the nuclear spent fuel reprocessing. Pt was impregnated in the catalysts via reductive heat treatment. The catalysts were characterized by x-ray diffraction, magnetization measurement, field emission scanning electron microscope and high-resolution transmission electron microscope studies. Catalysts reduced at 200 °C and above temperatures were found to be sufficient for the complete reduction of Pt to its Pt(0) state and were highly efficient for the U(IV) generation via hydrogenation. The U(IV) produced during the experiment was analyzed via the titrimetric and spectroscopic methods. Among all the catalysts prepared under the scope of the study, the highest saturation magnetization was measured for the Fe_3_O_4_-10SiO_2_/Pt catalysts prepared at 200 °C (Fe_3_O_4_-10SiO_2_/Pt(200)). However, the catalyst was least effective towards U(VI) reduction compare to the catalysts prepare at 250 °C (Fe_3_O_4_-10SiO_2_/Pt(250)) and 300 °C (Fe_3_O_4_-10SiO_2_/Pt(300)). The saturation magnetization of Fe_3_O_4_-10SiO_2_/Pt(300) was found to be higher than that of Fe_3_O_4_-10SiO_2_/Pt(250). Finally, Fe_3_O_4_-10SiO_2_/Pt(300) was considered a model catalyst for the detailed characterization, benchmarking and recycling of the catalyst material. Samples with higher silica content were also prepared at 300 °C and assessed for their catalytic activity.

**Supplementary Information:**

The online version contains supplementary material available at 10.1038/s41598-025-03867-y.

## Introduction

Nuclear power is considered the cheapest and a green source of energy towards grid stability as well as sustainability for heavy industries. Most of the currently operating thermal nuclear reactors (pressurized water reactor (PWR), Boiling water reactor (BWR), Pressurized heavy water reactor (PHWR)) in the world use < 5% of the initially loaded nuclear fuel materials for power generation; the remaining are discharged as spent fuel from the reactor. The spent fuels, discharged from those reactors, are reprocessed in nuclear fuel reprocessing plants to recover the valuable heavy metals, U and Pu^1,2^. As a part of long-term energy security, many countries are pursuing a fast reactor program to effectively utilize their nuclear material resources. These fast reactors require a very high percentage of fissile materials, mainly Pu, in their fuel element compared to the conventional reactor fuel element due to the very high neutron energy spectrum in these reactors^3–5^. However, most reactors consume only around 10 to 12% of the initial total heavy elements for power production. The rest is discharged again as spent fuel for reprocessing to close the fuel cycle. The reprocessing of these fast reactor spent fuels will no longer be the same as thermal reactor spent fuel reprocessing since the Pu content in these fuels, both from initial loading and breeding, is significantly higher than that of thermal reactor fuel. The spent fuel discharged from these fast reactors requires many innovative reprocessing steps to get back the U and Pu separated from the fission products^6,7^.

In the aqueous reprocessing of the fast reactor spent fuel (plutonium-uranium reduction extraction process), the spent fuel is dissolved in concentrated nitric acid, followed by co-extraction of U and Pu with tributyl phosphate (TBP)-dodecane from acidic aqueous phase. Subsequently, in the partitioning step, U and Pu are recovered individually from the extracted organic phase to the applicable level of purity. Due to the significant amount of Pu present in the fast reactor spent fuel, the partitioning of U and Pu poses challenges compared to that of U and Pu partitioning in PHWR spent fuel reprocessing. In the partitioning step, Pu(IV) is reduced to Pu(III), and TBP, having very little affinity for Pu(III), is back-extracted to the aqueous phase^7–9^. In the past, many reducing agents like ferrous sulphamate^10^, hydroxyl amine nitrate^11–13^, acetohydroxamic acid^14^ have been used for the reduction of Pu(IV). However, all these reducing agents have their own disadvantages. To overcome the disadvantages of the earlier reducing agents, U(IV) has been reported to be advantageous and is currently being used in the commercial reprocessing plants for reducing Pu(IV) to Pu(III) in the partitioning cycle^15,16^. Here, it is worth mentioning that U(IV) does not add any foreign element into the process stream again after the co-decontamination. However, since the Pu content in the fast reactor fuel is significantly high, a large quantity of U(IV) needs to be produced with high efficiency at a faster rate.

U(IV), which is the less familiar oxidation state of U in nitric acid medium, can be produced by reducing U(VI), either by electrochemically^17–21^, Photochemically^22,23^, photo-catalytically^24,25^ or catalytically^26–30^. The current method of electrochemical production of U(IV) offers certain advantages like ease of operation, reliability and simplicity. However, limitations like poor kinetics, lower efficiency and frequent maintenance of the electrode with a large volume of secondary waste generation limit its adaptation for large-scale production of U(IV)^29^. As an alternative to the electrochemical method, people have reported the catalytic method for U(IV) generation with faster kinetics and near 100% conversion of U(VI) to U(IV) to meet the demand for a large amount of U(IV). Novel metal, Pt, has been used as an active catalyst in many heterogeneous catalytic hydrogenation reactions because of the high stability of Pt in harsh reaction conditions. In recent literature, silica-supported Pts are also reported as an effective catalyst for the reduction of U(VI) to U(IV) in a highly corrosive aqueous nitric acid medium in the presence of hydrazine and/or under hydrogen^27–29,31^. However, it is worth noting that hydrazine is not always suitable as a reducing agent for the continuous production of U(IV) at a faster rate due to the slower kinetics compared to hydrogen^29^. At present, centrifugation is used as a sole technique to separate these catalysts from the reaction medium. The methodology requires additional processes and high-maintenance equipment to recover this catalyst for its continuous recycling.

To address those issues, in this work, we have developed a series of highly efficient magnetically separable Fe_3_O_4_ incorporated SiO_2_/Pt catalysts (Fe_3_O_4_-SiO_2_/Pt) and investigated the efficiency of the catalysts for the reduction of U(VI) to U(IV) using hydrogen and compared them with the literature reported catalysts. The introduction of magnetic nanoparticles in the catalyst facilitates its separation from the medium by applying an external magnetic field without compromising the catalytic efficiency of the material^32,33^. The present report is a first-of-a-kind investigation that uses a magnetic nanoparticle-incorporated catalyst for U(IV) production.

## Materials and methods

### Materials

The chemicals used for the experiment were procured from the indicated vendors. Ferric chloride (FeCl_3_), ferrous chloride (FeCl_2_) and tetraethyl orthosilicate (TEOS) were procured from Alfa Aesar. Hydrochloric acid (HCl), sulfuric acid (H_2_SO_4_), nitric Acid (HNO_3_) and hydrazine hydrate (N_2_H_4_. H_2_O) were procured from Merck. Ethanol was procured from Hayman Ltd. Ammonium hydroxide (NH_4_OH) was purchased from Sisco Research Laboratories Pvt. Ltd. Chloroplatinic acid hexahydrate (H_2_PtCl_6_.6H_2_O) and ferroin indicator were procured from Sigma-Aldrich. All these chemicals were of analytical grade and were used directly without further purification. Hydrogen gas (Purity: 99.99%) used in the experiments was supplied by Atmospheric Speciality Gases Private Limited, Ahmedabad, India. Uranium for the experiments was sourced from the Nuclear Fuel Complex, Hyderabad, India, in the form of UO_2_ pellets and used after dissolving in concentrated HNO_3_. Deionised water obtained from a Millipore water system was used for all the experiments. A neodymium magnet of width 4 cm and height 1 cm (field strength ~ 100 gauss at 1 cm) was used to recover the catalyst material after the reduction reaction.

### Synthesis

#### Synthesis of Fe_3_O_4_–xxSiO_2_ particles

A typical synthesis scheme is as follows. At first, Fe_3_O_4_ nanoparticles were synthesized via the co-precipitation method as reported elsewhere^34^. In brief, 8 mL 1 (M) FeCl_3_ and 2 mL 2 (M) FeCl_2_ solutions were taken in a beaker and added to a 100 mL 0.7 (M) NH_4_OH solution. The mixture was then stirred for 30 min with a mechanical stirrer in order to obtain Fe_3_O_4_ nanoparticles. Then, this product was separated from the solution with the help of an external magnetic field and was washed several times with water. Subsequently, 50 mL 2 (M) HCl was added to these particles and sonicated for 10 min to enable coating of Fe_3_O_4_ particles with SiO_2_. Then, the supernatant was decanted, and 100 mL 80% ethanol-water mixture was added to form a stable suspension. Finally, 2 mL concentrated NH_4_OH was added to this suspension, followed by 10 mL TEOS, which was added dropwise to the reaction mixture from a pressure equilibration funnel under continuous stirring. Stirring was continued for about 16 h in order to obtain the Fe_3_O_4_−10SiO_2_^35^. Similarly, all other silica-containing samples were synthesized by increasing the amount of TEOS in the reaction medium.

#### Synthesis of Fe_3_O_4_–xxSiO_2_/Pt(xxx)

1 g of dry Fe_3_O_4_−10SiO_2_ powder was taken in a 100 mL round-bottom flask, and 50 mL of ethanol was added to it. After 5 min of sonication, 1.4 mL of 77 mM chloroplatinic acid was added to this suspension, and the latter was stirred for 24 h before the solvent was evaporated and the dry powder was obtained. The dried powder was placed in a tubular furnace and heated at 150, 200, 250 and 300 °C under 8% H_2_-Ar with a flow rate of 100 mL/min for 3 h. in order to obtain the Fe_3_O_4_−10SiO_2_/Pt(150), Fe_3_O_4_−10SiO_2_/Pt(200), Fe_3_O_4_−10SiO_2_/Pt(250) and Fe_3_O_4_−10SiO_2_/Pt(300), respectively. For the synthesis of higher silica-containing, Fe_3_O_4_−15SiO_2_/Pt(300), Fe_3_O_4_−20SiO_2_/Pt(300) and Fe_3_O_4_−25SiO_2_/Pt(300), a similar procedure was followed and directly heat-treated at 300 °C under an 8% H_2_-Ar at a flow rate of 100 mL/min for 3 h. The details of all the samples synthesized under the scope of the study are summarised in Table S1 along with the synthetic conditions.

### Characterization techniques

Platinum content in all these samples was determined by using inductively coupled plasma optical emission spectroscopy (ICP-OES; Spectro Arcos, Germany). In a typical analysis, a known weight of the catalyst was dissolved in a known volume of aqua regia, and this solution was subjected to ICP-OES analysis. The samples were characterized for their constituent phase by using powder X-ray diffraction (XRD; STOE) with Cu Kα radiation with a 0.1° step size and a 15 s data acquisition time. The morphology of the samples was evaluated using the FESEM images, recorded in a Zeiss Ultra Plus FESEM microscope, which was equipped with an Energy-dispersive X-ray spectroscopy (XEDS) detector (Oxford Instruments). The FESEM images were recorded by taking a small amount of powder sample over a carbon tape, fixed to the sample holder. Further, transmission electron microscope (TEM), high-resolution transmission microscopy (HRTEM) and high-angle annular dark-field scanning transmission electron microscope (HAADF-STEM) images for Fe_3_O_4_−10SiO_2_/Pt(300) were recorded using a ThermoFisher make Talos 200 FS TEM. Elemental mapping data in STEM mode were recorded using a 2-quadrant XEDS detector. Magnetization was measured with a vibrating sample magnetometer (Lakeshore, Model 7407). UV-Vis spectra were recorded using a Thermo Scientific Evolution One Plus UV-Vis spectrophotometer. Fourier transform infrared spectra (FTIR) were recorded in attenuated total reflection (ATR) mode using BRUKER alpha II FTIR spectrophotometer. X-ray photoelectron spectra (XPS) were recorded using monochromatic Al Kα emission in Thermo Scientific K-Alpha instrument. BET surface area measurements were carried out by using the N_2_ adsorption-desorption technique in a Micromeritics ASAP 2020 porosimeter instrument.

### Uranium reduction studies

Uranium reduction studies were carried out by taking 0.25 (M) of U(VI) solution in 1.3 (M) HNO_3_, containing hydrazine to the strength of 0.2 (M) as a stabilizing agent for U(IV). Initially, 200 mL of U(VI) solution was taken in a 450 mL SS304 autoclave supplied by Amar Equipment Pvt. Ltd, Mumbai. A hydrogen pressure of 2 bar was maintained inside the reactor using a pressure control valve, and the solution was stirred with a motorized external stirrer at 1200 rpm. In all the experiments, a Pt catalyst to U atomic ratio was maintained at 1:2000, except otherwise mentioned.

**Fig. 1:**
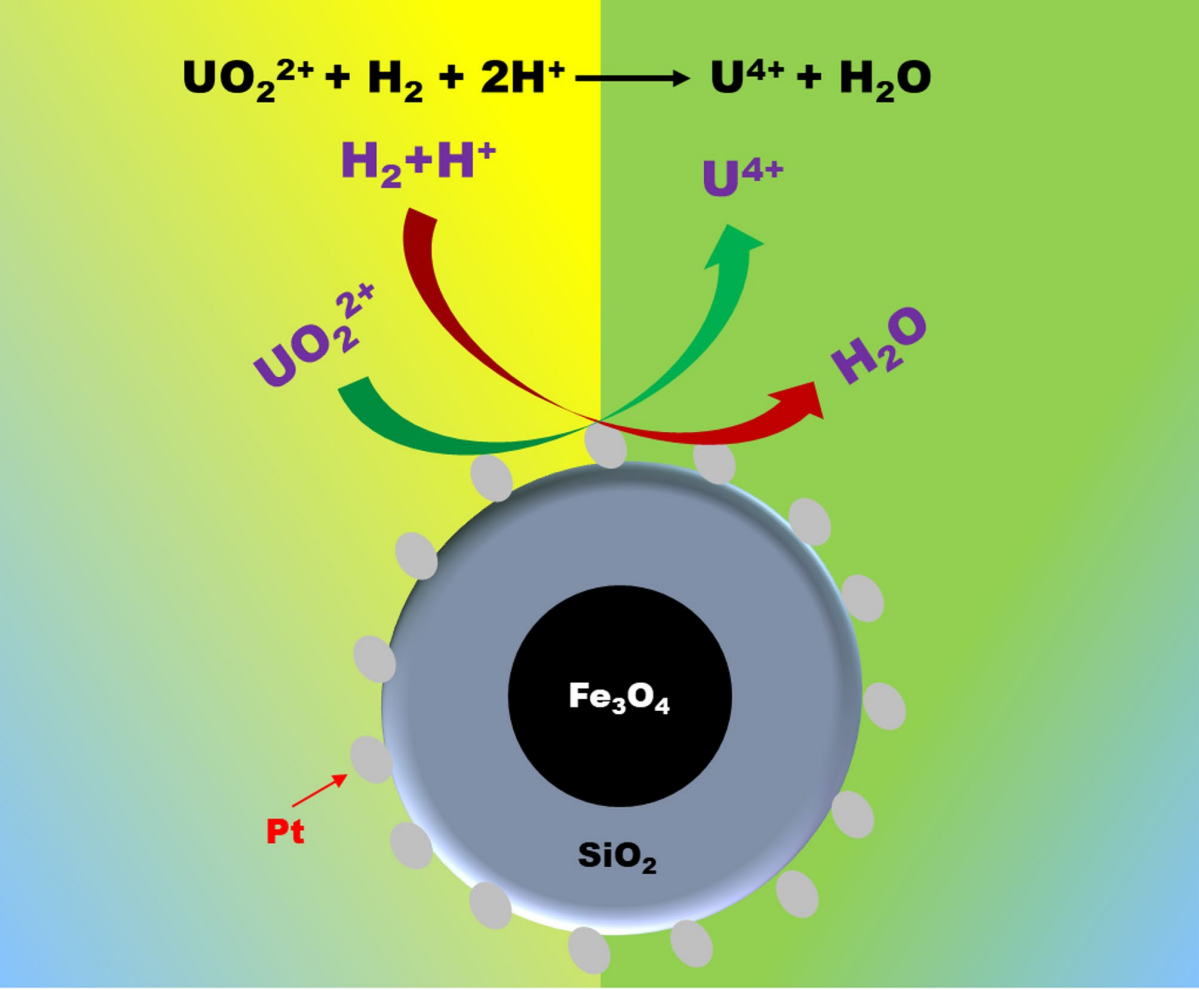
Schematic of U(VI) reduction over Fe_3_O_4_-SiO_2_/Pt catalysts.

Liquid samples were taken out at regular fixed time intervals through a dip nozzle, provided at the top of the autoclave, by controlling the gas pressure inside. After centrifugation, a desired amount of the aliquot was taken and titrated against potassium dichromate in ~ 2 (M) H_2_SO_4_ medium in the presence of a ferroin indicator. UV-Vis spectra of the aliquots collected at a fixed time interval were also recorded for the kinetic experiment with Fe_3_O_4_−10SiO_2_/Pt(300) as catalysts. Figure 1 shows the schematic for the U(VI) reduction over Fe_3_O_4_-SiO_2_/Pt catalysts.

## Results & discussion

### Mechanism involved in the synthesis

Initially, Fe_3_O_4_ nanoparticles were synthesized by taking a mixture of Fe (III) and Fe(II) in the proportion 2:1 and adding the latter to NH_4_OH medium. NH_4_^+^ ions protect the Fe_3_O_4_ particles synthesized in this way and possess a positive surface charge. The positive surface charge on the Fe_3_O_4_ particles does not allow SiO_2_ to grow over this surface; nucleation and growth of SiO_2_ happen separately in the medium to form a separate white SiO_2_ phase distinct from the black colour Fe_3_O_4_ particles.

However, once the Fe_3_O_4_ particles are treated with HCl, Cl^−^ ions replace the NH_4_^+^ ions on the surface of Fe_3_O_4_, making the surface charge negative and ready for dispersion in ethanol-water medium, suitable for coating it with SiO_2_^36^. Hence, treating these particles with HCl before coating them with SiO_2_ is essential.

### Pt estimation

The platinum content in the samples estimated by ICP-OES was found to be in the range of 1.7 to 1.8 weight percentages. The analytical results are presented in the supplementary document (Table S1).

### Crystallographic structural studies

**Fig. 2:**
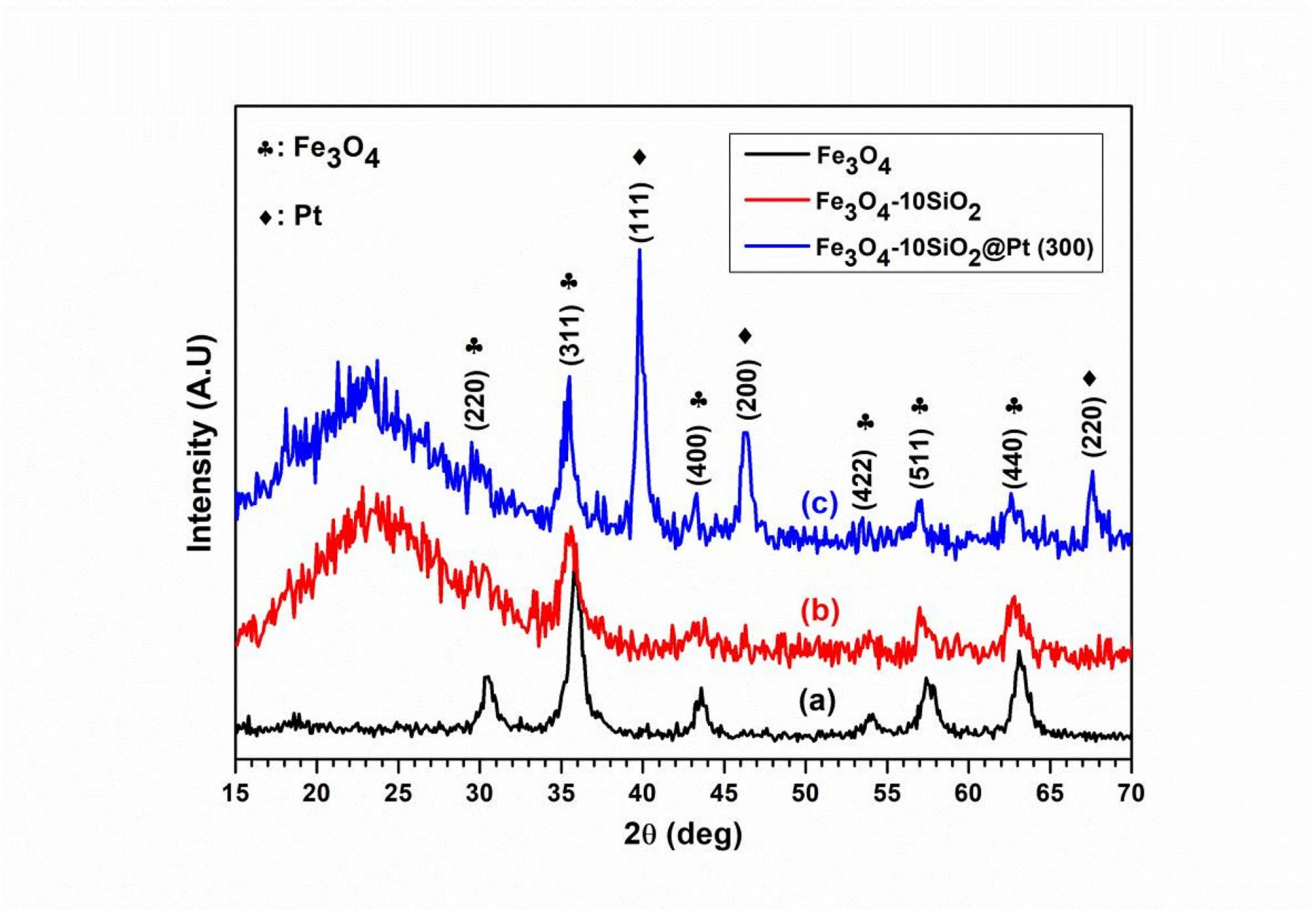
XRD Pattern of Fe_3_O_4_ (**a**), Fe_3_O_4_−10SiO_2_ (**b**) and Fe_3_O_4_−10SiO_2_/Pt(300) (**c**).

The XRD patterns of Fe_3_O_4_, Fe_3_O_4_−10SiO_2_ and Fe_3_O_4_−10SiO_2_/Pt(300) are shown in Fig. 2. The XRD pattern (a) in Fig. 2 shows the peaks corresponding to Fe_3_O_4_ phase. The peaks at 2θ values of 29.95, 35.47, 43.24, 57.12 and 62.91° correspond to (200), (311), (400), (511) and (440) planes of Fe_3_O_4_, respectively (JCPDS or ICCD card # 19–0629). These XRD patterns reveal no peaks corresponding to any other iron oxide phase. It may be noted that γ-Fe_2_O_3_ possesses a similar crystal structure to Fe_3_O_4_ with accelerated Fe(II) vacancies. The data from the Fe_3_O_4_ XRD pattern was used to calculate the particle size of Fe_3_O_4_ using the Scherrer equation. The particle size of Fe_3_O_4_ was calculated to be 9.53(± 1.30) nm. The XRD pattern of Fe_3_O_4_−10SiO_2_ ((b) in Fig. 2) shows the presence of the Fe_3_O_4_ phase along with the presence of a broad peak at 2θ in the range of 15° to 30°, which is ascribed to the amorphous SiO_2_. The plot (c) in Fig. 2, corresponding to the XRD pattern of Fe_3_O_4_−10SiO_2_/Pt(300), shows additional peaks corresponding to pure metallic platinum (*hkl*) planes of (111), (200) and (220) appearing at 2θ values of 39.73°, 46.30° and 67.58°, respectively (JCPDS or ICCD card # 00–004-0802).

The XRD pattern after impregnation of H_2_PtCl_6_. 6H_2_O, followed by reduction at 150 °C under a flowing stream of 8% H_2_-Ar atmosphere for three hours, shows several peaks corresponding to the PtCl_4_ phase, indicating that the reduction is incomplete at 150 °C. However, when the reduction temperature was increased to 200 °C and above, XRD peaks corresponding to pure metallic platinum appeared (Figure S1). Further, the intensities of the Bragg reflections pertaining to the Fe_3_O_4_ phase peak were found to decrease with the increase of SiO_2_ loading, which masks the Fe_3_O_4_ particles from being probed with X-ray (Figure S2). The XRD pattern of all higher silica-containing catalysts prepared at 300 °C also shows the peak corresponding to metallic Pt (Figure S3).

### Morphological and compositional studies

**Fig. 3:**
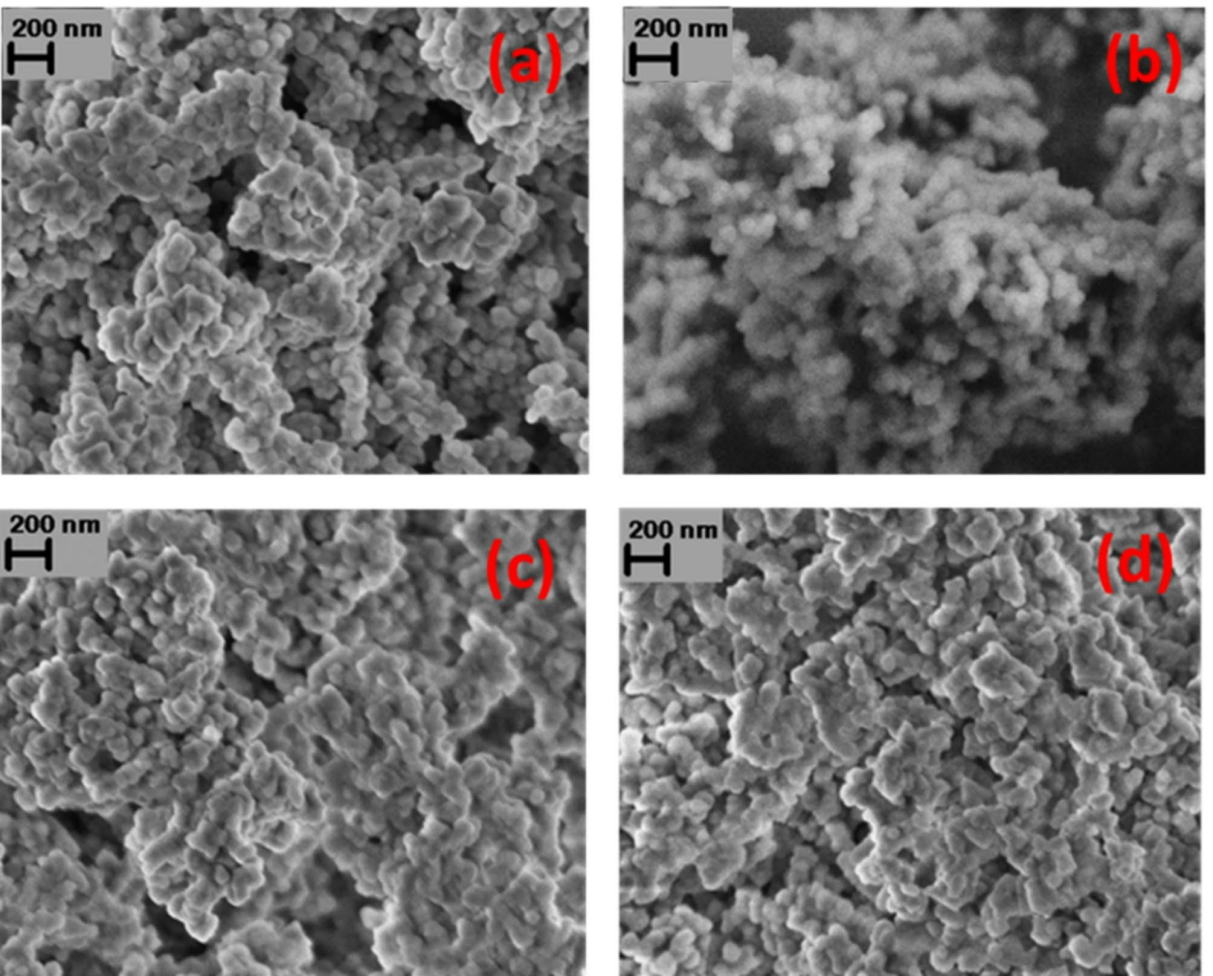
FESEM image of Fe_3_O_4_−10SiO_2_ (**a**), Fe_3_O_4_−10SiO_2_/Pt(200) (**b**), Fe_3_O_4_−10SiO_2_/Pt(250) (**c**) and Fe_3_O_4_−10SiO_2_/Pt(300) (**d**).

Figure 3 shows the FESEM image of (a) Fe_3_O_4_−10SiO_2_ and (b) Fe_3_O_4_−10SiO_2_/Pt(200) (c) Fe_3_O_4_−10SiO_2_/Pt(250) (d) Fe_3_O_4_−10SiO_2_/Pt(300) catalysts. Agglomerated spherical particles with identical morphology were observed in all samples. The energy dispersive X-ray (XEDS) analysis confirmed the presence of Pt in Fe_3_O_4_−10SiO_2_/Pt(200), Fe_3_O_4_−10SiO_2_/Pt(250) and Fe_3_O_4_−10SiO_2_/Pt(300) (Figure S4-S6). With the increased SiO_2_ content in the samples, an increasing trend in particle size was observed with higher agglomeration (Figure S7). The BET surface area measured for Fe_3_O_4_−10SiO_2_ and Fe_3_O_4_−10SiO_2_/Pt(300) was found to be 25.18 m^2^/g and 21.97 m^2^/g, respectively, and no significant pore structures were detected during the N_2_ adsorption-desorption measurement (Figure S8-9).

**Fig. 4:**
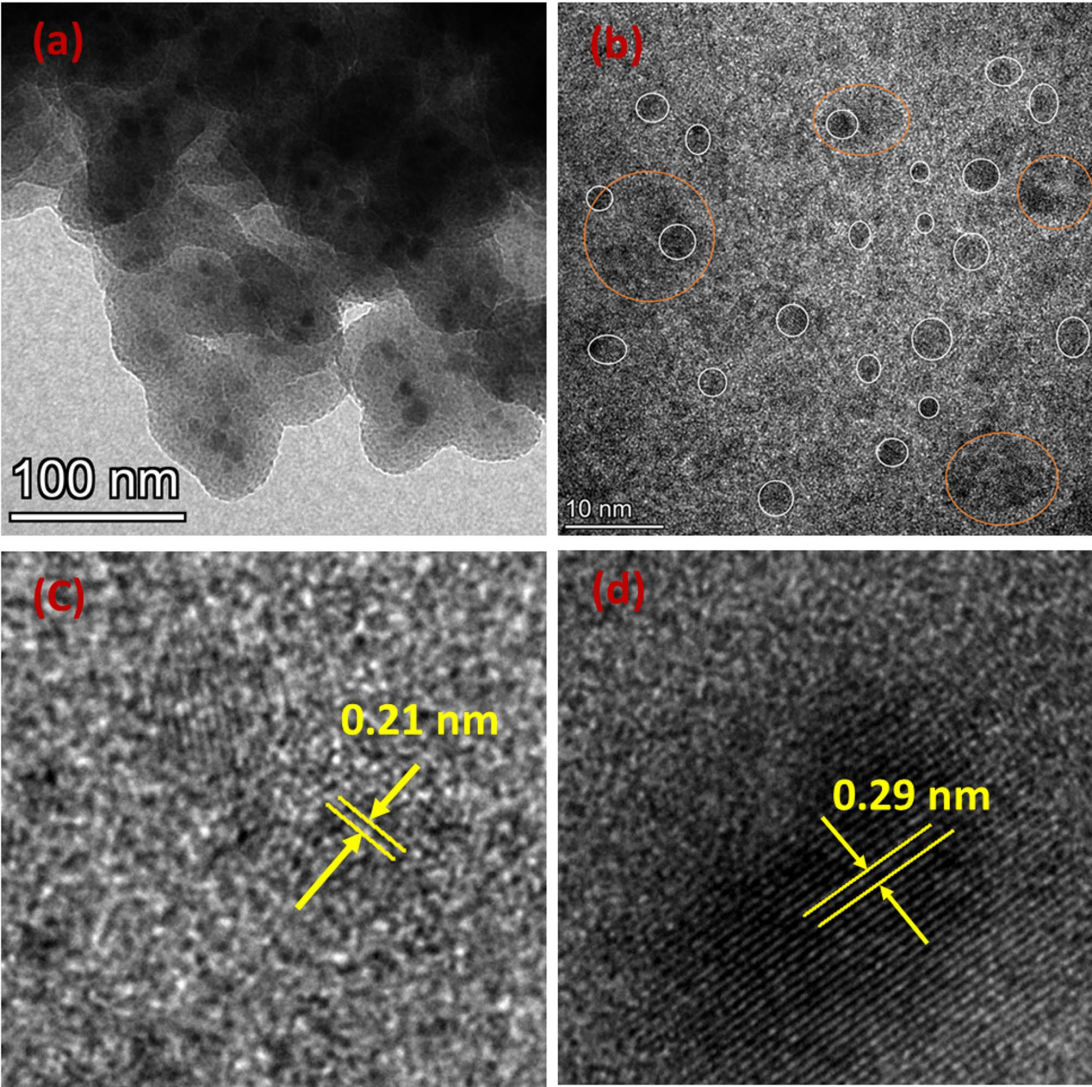
TEM image of Fe_3_O_4_−10SiO_2_/Pt(300) (**a**,**b**) (small white circles show the Pt nanoparticles and bigger orange circles show the Fe_3_O_4_ particles), HRTEM image of the Pt nanoparticles (**c**) and Fe_3_O_4_ particle (**d**).

**Fig. 5:**
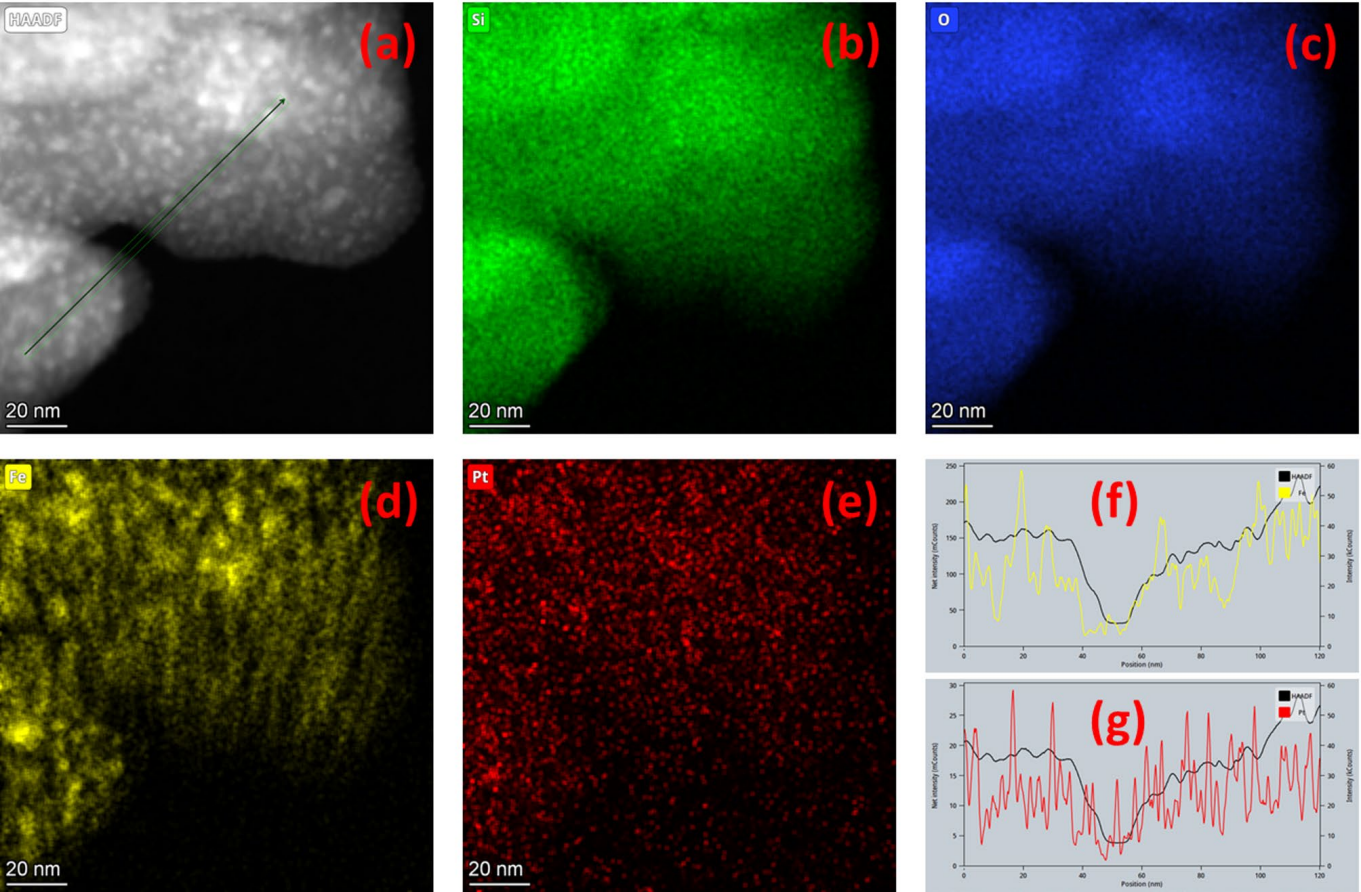
HAADF-STEM image of Fe_3_O_4_−10SiO_2_/Pt(300) (**a**). Elemental mapping image of Si (**b**), O (**c**), Fe (**d**) and Pt (**e**). Line profile of Fe (**f**) and Pt (**g**) with reference to the HAADF-STEM image (**a**).

Further, the TEM image for Fe_3_O_4_−10SiO_2_/Pt(300) clearly shows the embedded Fe_3_O_4_ within the agglomerated SiO_2_ particles with finely distributed Pt over the material (Fig. 4a, b). TEM image reveals that the diameter of the Pt nanoparticles over the Fe_3_O_4_−10SiO_2_ matrix is in the range of 2 to 4 nm, with ~ 10 nm diameter Fe_3_O_4_ particles. The HRTEM of the Pt particles shows the lattice spacing of 0.21 nm, corresponding to the (111) plane of metallic Pt (Fig. 4c)^37^. Figure 4d shows the HRTEM image of the Fe_3_O_4_ particle in the SiO_2_ matrix with a lattice spacing of 0.29 nm, which corresponds to (220) plane of Fe_3_O_4_^38^. HAADF-STEM and XEDS elemental mapping images (Fig. 5a-e) reveal that the Pt nanoparticles are very finely distributed over the matrix, whereas bigger Fe_3_O_4_ particles are present within the matrix at a larger distance. To clarify further, the line profile was also drawn with reference to the HAADF-STEM image (Fig. 5f-g). The line profiles for both Fe and Pt are consistent with the HAADF profile, confirming the presence of both elements within the matrix. Both the Fe and Pt follow a zigzag intensity profile, depicting the particle nature of both Fe_3_O_4_ and Pt. However, the intensity peaks are much sharper and more closely spaced in the case of Pt as compared to the broader and largely spaced intensity peak for Fe, also indicating the much finer particle size and more homogeneous distribution of Pt particles over the matrix as compared to the embedded Fe_3_O_4_ particles.

### Magnetization study

The magnetization of all the samples was measured at room temperature, and the variation of the same with field strength showed that all these samples exhibit superparamagnetic behavior at room temperature with no residual magnetic moment at zero field strength. This phenomenon is also consistent with the XRD pattern of Fe_3_O_4_ and the crystallite size (9.3 nm) calculated from the XRD pattern using the Scherrer equation, which is well below the superparamagnetic size of Fe_3_O_4_, ~ 25 nm^39^The materials’ superparamagnetic behavior is essential for our application since the residual magnetic moment may hinder the particles’ re-dispersion in the subsequent step once they are recovered from the reaction medium with the help of an external magnet.

**Fig. 6:**
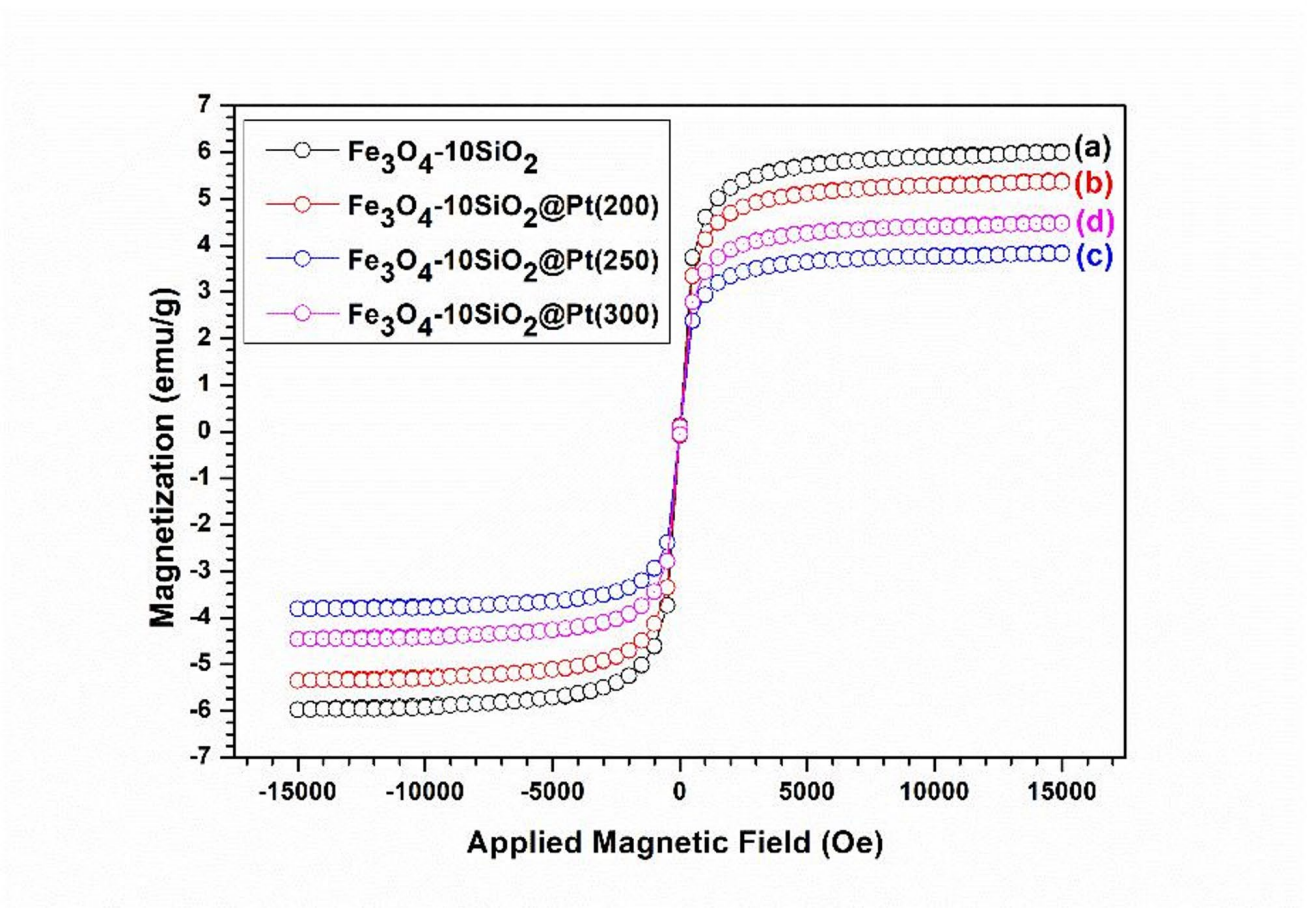
Variation in the magnetization with applied magnetic field of Fe_3_O_4_−10SiO_2_ (**a**), Fe_3_O_4_−10SiO_2_/Pt (200) (**b**), Fe_3_O_4_−10SiO_2_/Pt (250) (**c**) and Fe_3_O_4_−10SiO_2_/Pt (300) (**d**).

In the magnetization studies for Fe_3_O_4_−10SiO_2_, Fe_3_O_4_−10SiO_2_/Pt(200), Fe_3_O_4_−10SiO_2_/Pt(250) and Fe_3_O_4_−10SiO_2_/Pt(300), the order of magnetization was found to be, Fe_3_O_4_−10SiO_2_ > Fe_3_O_4_−10SiO_2_/Pt(200) > Fe_3_O_4_−10SiO_2_/Pt(300) > Fe_3_O_4_−10SiO_2_/Pt(250) (Fig. 6). The saturation magnetization decreases with the loading of Pt particles in all the catalysts, which in turn reduces the Fe_3_O_4_ percentage in the final catalyst material. The highest saturation magnetization value of 4.8 emu/g among the Pt loaded catalysts observed with the sample reduced at 200 °C may be attributed to the least deteriorated Fe_3_O_4_ phase at 200 °C. The Fe_3_O_4_ phase starts deteriorating with increasing reduction temperature, and magnetization decreases. The increase in magnetization for the sample reduced at 300 °C compared to the sample reduced at 250 °C may be attributed to the evaporation of the non-magnetic volatile components from the sample at higher temperature. With reasonable magnetization, the Fe_3_O_4_−10SiO_2_/Pt(300) catalyst is used as a model system for other subsequent measurements.

The Fe_3_O_4_−10SiO_2_/Pt(300) catalyst with the highest proportion of magnetic Fe_3_O_4_ also showed a high value of magnetization as compared to other samples with higher silica content. The order of magnetization follows Fe_3_O_4_−10SiO_2_/Pt(300) > Fe_3_O_4_−15SiO_2_/Pt(300) > Fe_3_O_4_−20SiO_2_/Pt(300) > Fe_3_O_4_−25SiO_2_/Pt(300) (Figure S10).

### Uranium reduction studies

**Fig. 7:**
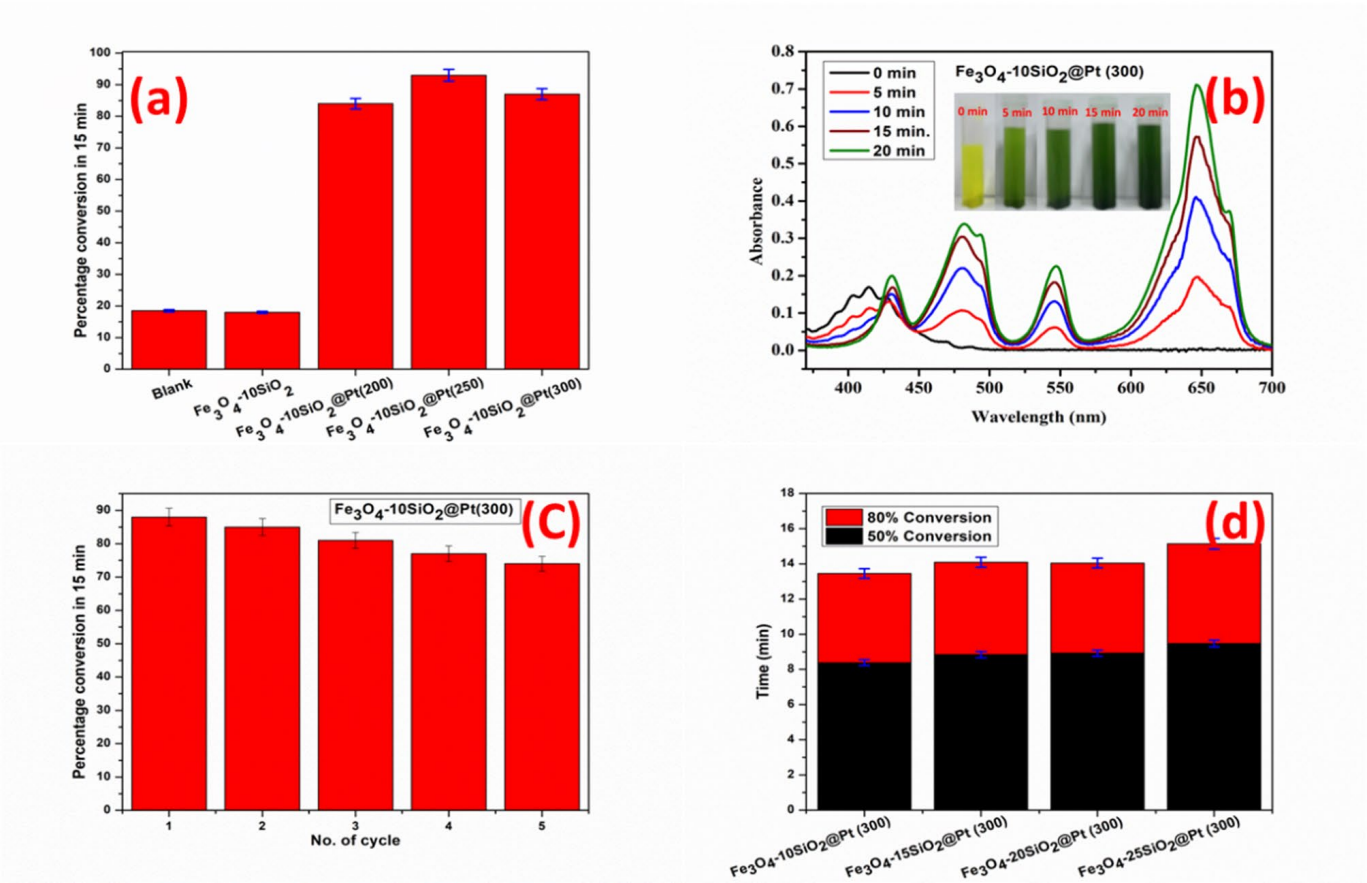
Percentage conversion of U(VI) to U(IV) in 15 min for Fe_3_O_4_−10SiO_2_/Pt catalysts with reference to blank and Fe_3_O_4_−10SiO_2_ (**a**), UV-Vis spectra of uranium solution at different time intervals during the reduction using Fe_3_O_4_−10SiO_2_/Pt(300) catalyst. Inset show the digital photograph of U(IV) solution at different time interval (**b**). Percentage conversion of U(VI) to U(IV) in 15 min for Fe_3_O_4_−10SiO_2_/Pt(300) catalysts up to five consecutive cycles (**c**), bar plot of up to 80% U(VI) to U(IV) conversion for catalysts with different proportion of SiO_2_, synthesized at 300 °C (**d**).

Figure 7a shows the percentage of U(VI) converted to U(IV) in 15 min in case of no catalyst (blank), Fe_3_O_4_−10SiO_2_, Fe_3_O_4_−10SiO_2_/Pt(200), Fe_3_O_4_−10SiO_2_/Pt(250) and Fe_3_O_4_−10SiO_2_/Pt(300) samples. It is evident that the matrix (Fe_3_O_4_-xxSiO_2_) does not play any catalytic role for the U(VI) to U(IV) conversion under hydrogen, since only ~ 18% conversion was achieved in both the cases of blank and Fe_3_O_4_−10SiO_2_ sample for the same time period. However, when Pt was incorporated into the materials, the reaction kinetics accelerated, and ≥ 84% conversion was achieved within 15 min from the start of the reaction in all the cases (Corresponding raw data plots are available in the supplementary file; figures S11-S15). It might be noted that ~ 65 min was required to achieve the same in the case of blank and using only Fe_3_O_4_−10SiO_2_. All the three samples Fe_3_O_4_−10SiO_2_/Pt(200), Fe_3_O_4_−10SiO_2_/Pt(250) and Fe_3_O_4_−10SiO_2_/Pt(300) were proven to be highly effective towards the conversion of U(VI) to U(IV) under the said condition with the order of performance Fe_3_O_4_−10SiO_2_/Pt(250) > Fe_3_O_4_−10SiO_2_/Pt(300) > Fe_3_O_4_−10SiO_2_/Pt(200). However, the magnetic property of Fe_3_O_4_−10SiO_2_/Pt(250) was found to be the lowest as compared to Fe_3_O_4_−10SiO_2_/Pt(200) and Fe_3_O_4_−10SiO_2_/Pt(300), as discussed earlier (Fig. 6). No U(VI) reduction experiment was conducted with the sample reduced at 150 °C, since XRD confirmed that the complete reduction of the chloroplatinic acid to metallic Pt had not occurred in this sample.

Further experiments were carried out to test the recyclability of the catalysts using Fe_3_O_4_−10SiO_2_/Pt(300) model catalyst under the same conditions. Figure 7b shows the UV-Vis spectra of the U solution at different time intervals during the first U(VI) reduction cycle. With time, the absorbance for U(IV) at 430 nm, 481 nm, 564 nm and 647 nm has increased, whereas the absorbance for U(VI) at 414 nm has decreased with an isobestic point near 425 nm, indicating the conversion of U(VI) to U(IV) with the progress of the reaction^40^. The data presented in Fig. 7c up to 5 cycles reveals that the catalytic performance gradually decreases with the increase of the number of cycles, partly of which is attributed to the loss of the catalysts from incomplete recovery for the consecutive cycle.

The activation energy for the reaction calculated from the initial rate parameter based on the experiment performed at different temperatures was found to be 21.12 kJ/mol, which is in good agreement with our earlier reported value using SiO_2_/Pt catalyst^41^. Based on our previous studies with SiO_2_/Pt catalysts, here also we propose, Fe_3_O_4_-SiO_2_/Pt catalyst undertake similar mechanism towards reducing U(VI) to U(IV) in HNO_3_-N_2_H_4_ medium, since in both the cases Pt only acts as an active surface towards catalytic reduction of U(VI) to U(IV). In brief, the catalytic reduction of U(VI) by H_2_ over Pt takes place, where initially a molecularly adsorbed U(VI), HNO_3_ over the catalyst surface interacts with the atomically adsorbed hydrogen. In this mechanism, the adsorption of HNO_3_ on the catalyst surface determines the rate of the reaction.

Finally, the U(VI) to U(IV) reduction experiments were carried out with all the higher silica-containing catalysts synthesized at 300 °C, Fe_3_O_4_−15SiO_2_/Pt(300), Fe_3_O_4_−20SiO_2_/Pt(300) and Fe_3_O_4_−25SiO_2_/Pt(300) to assess the role of the matrix. Figure 7(d) summarizes the results in terms of time taken for the U(VI) to U(IV) conversion up to 50% and 80% for those catalysts. The decrease in catalytic activity with the increase of silica content is attributed to the lesser available surface area in those samples due to the increase in particle size with the increase in silica content (Corresponding raw data plots are available in the supplementary file, figures S16-S18). It is also worth mentioning that in all the cases, a complete 100% conversion was achieved in less than 20 min, where Pt was used as an active surface for the catalytic conversion. The benchmark experiments were carried out under identical experimental conditions as reported in the literature^29,30,41^. The results are summarized in the supplementary information Table-S2. In all the cases the catalysts have outperformed as compared to those reported catalysts.

### Post-application characterization

**Fig. 8:**
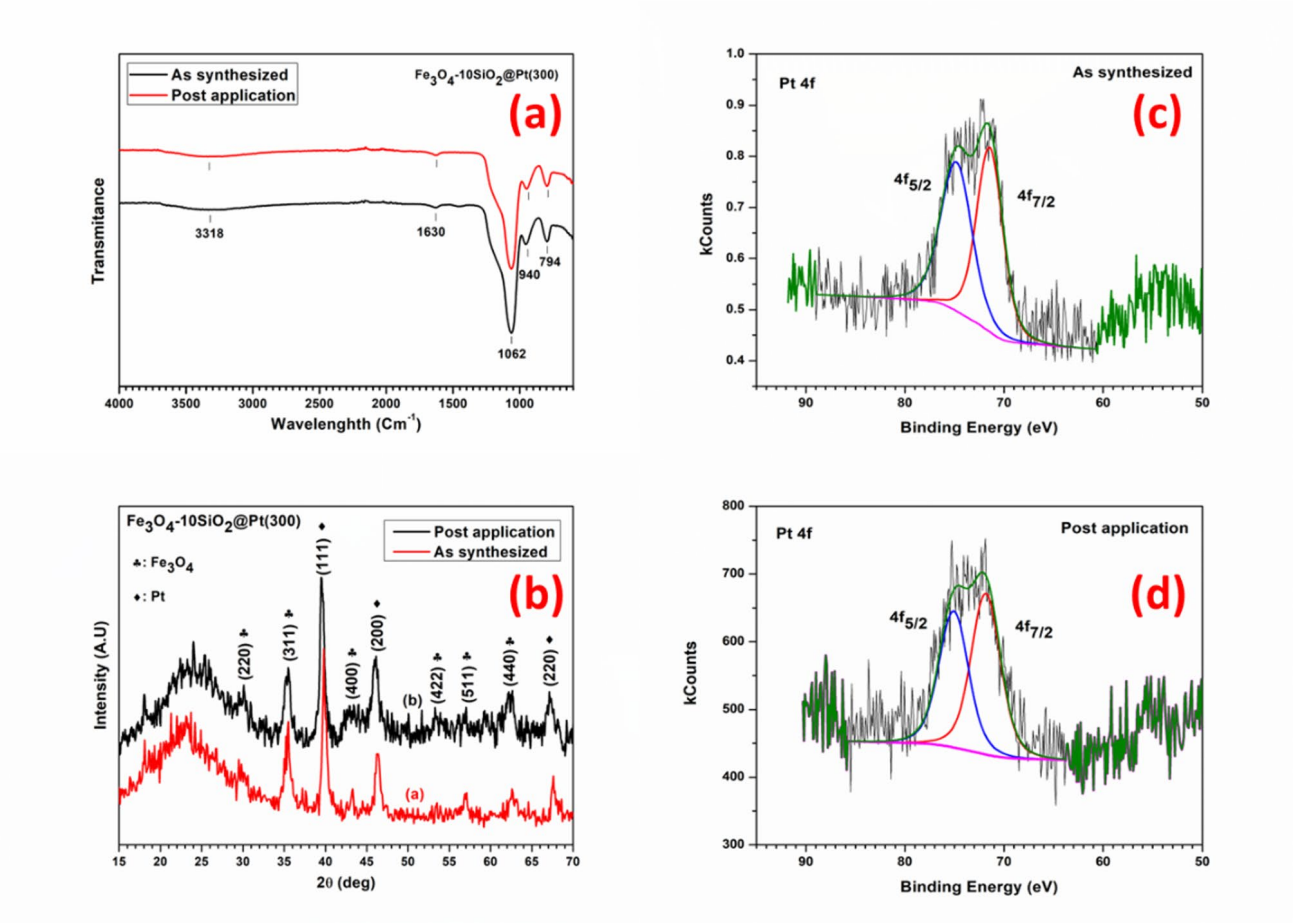
Comparative FTIR (**a**), XRD (**b**) and XPS (**c**,**d**) spectra of Fe_3_O_4_−10SiO_2_/Pt(300) samples, before and after the application for U(VI) reduction.

A post-application (for 5 consecutive cycles) characterization of the Fe_3_O_4_−10SiO_2_/Pt(300) catalysts using FTIR, XRD and XPS studies reveals that the synthesized catalyst is highly stable in the reaction medium and there are no significant changes in the catalyst’s chemical, crystal and electronic structures as can be probed by these techniques. The FTIR spectra of the samples (Fig. 8a) recorded in the range of 4000 to 600 cm^−1^, before and after the application, show that the silica structure in the sample remains intact with a major peak at 1062 cm^−1^ corresponding to Si-O-Si vibration present in both the samples. The other peaks at 940 cm^−1^ and 794 cm^−1^ correspond to Si-OH and Si-O-Si vibration, respectively, and are also present in both samples. The peaks at 3318 cm^−1^ and 1630 cm^−1^ correspond to the absorbed water molecules in the samples. The XRD pattern recorded for the used sample shows no significant changes in the crystal structure of Fe_3_O_4_ and Pt in the catalyst material (Fig. 8b). The XPS studies carried out to understand the Pt electronic state in the catalyst before and after the application revels that in both the samples Pt oxidation states nearly matches with the Pt(0) state with 4f_7/2_ binding energy being 71.4 eV and 71.8 eV respectively and 4f_5/2_ binding energy being 74.8 eV and 75.1 eV respectively (Fig. 8c-d). The little increase in Pt 4f binding energy in the used sample may be attributed to the partial loss of the electron from the Pt surface during the U(VI) reduction reaction.

## Conclusion

In summary, a method has been developed for synthesizing a magnetic silica-supported platinum catalyst, and the synthetic conditions are optimized. The catalysts were deployed for uranium reduction under a hydrogen atmosphere after detailed characterization of the as-synthesized catalyst for their morphologies, compositions, and crystallographic and chemical structures. All the catalysts synthesized at 200 °C and above have been found to be highly efficient compared to all earlier reported catalysts in the literature for U(VI) to U(IV) conversion. The Fe_3_O_4_−10SiO_2_/Pt(300) used as a model catalyst showed the optimum performance for the U(VI) to U(IV) conversion with a high degree of magnetization. The recyclability study and post-application characterizations confirm that the material possesses catalytic activity up to several cycles for reuse. With the increase of SiO_2_ content in the material, the catalyst performance slightly decreased due to the less available surface area for Pt impregnation, owing to the bigger particle size in those samples.

## Electronic supplementary material

Below is the link to the electronic supplementary material.

Supplementary Material 1

## Data Availability

All data are available in the article or additional information file.
