# Supplementary material for: Magnetically separable Fe3O4-SiO2/Pt catalyst and its application for uranium reduction
Source: Sci Rep. 2025 Jul 4;15:23853. doi: 10.1038/s41598-025-03867-y (PMC12227647; doi:10.1038/s41598-025-03867-y)
Supplement: Supplementary file 1 — Supplementary Material 1 [file 41598_2025_3867_MOESM1_ESM.docx]

**Development of Magnetically Separable Fe_3_O_4_-SiO_2_/Pt Catalyst and its Application for Uranium Reduction**

Kuntal Kumar Pal^1, 2 *^, Ramakrishna Reddy^2, 3^, Chanchal Ghosh^4^, K Ananthasivan ^5^, P. Velavendan^1^, Ramesh L. Gardas^6^, Sandip Dhara^2, 7 *^

^1^ Reprocessing Material Development Section, Process Radiochemistry Reprocessing Research & Development Division, Reprocessing Group, Indira Gandhi Centre for Atomic Research, Kalpakkam- 603102

^2^ Homi Bhabha National Institute, Anushaktinagar, Mumbai- 400094

^3^ Minor Actinide Chemistry and Reconversion Section, Process Radiochemistry Reprocessing Research & Development Division, Reprocessing Group, Indira Gandhi Centre for Atomic Research, Kalpakkam- 603102

^4^ Physical Metallurgy Division, Metallurgy & Materials Group, Indira Gandhi Centre for Atomic Research, Kalpakkam- 603102

^5^ Reprocessing Group, Indira Gandhi Centre for Atomic Research, Kalpakkam- 603102

^6^ Department of Chemistry, Indian Institute of Technology, Chennai- 600036

^7^ Material Science Group, Indira Gandhi Centre for Atomic Research, Kalpakkam- 603102

Email: [kuntal/igcar.gov.in](mailto:kuntal@igcar.gov.in); [dhara](mailto:dhara)/igcar.gov.in

**Table S1.** Details of the major synthetic parameters for the samples

| **Sample Name** | **Fe_3_O_4_**  **(g)** | **TEOS**  **(mL)** | **Fe_3_O_4_:SiO_2_**  **(Mole ratio)** | **H_2_PtCl_6_.6H_2_O**  **(77 mM)**  **(mL)** | **Reduction Temp**  **(8%H2-Ar)**  **(°C)** | **Final Pt Content**  **(Wt.%)**  **(As per ICP-OES)** |
| --- | --- | --- | --- | --- | --- | --- |
| Fe_3_O_4_-10SiO_2_ | 0.93 | 10 | 1:11.25 | --- | --- | --- |
| Fe_3_O_4_-15SiO_2_ | 0.93 | 15 | 1:16.92 | --- | --- | --- |
| Fe_3_O_4_-20SiO_2_ | 0.93 | 20 | 1:22.50 | --- | --- | --- |
| Fe_3_O_4_-25SiO_2_ | 0.93 | 25 | 1:28.20 | --- | --- | --- |
| Fe_3_O_4_-10SiO_2_/Pt(150) | 0.93 | 10 | 1:11.25 | 1.4 | 150 | --- |
| Fe_3_O_4_-10SiO_2_/Pt(200) | 0.93 | 10 | 1:11.25 | 1.4 | 200 | 1.75 |
| Fe_3_O_4_-10SiO_2_/Pt(250) | 0.93 | 10 | 1:11.25 | 1.4 | 250 | 1.73 |
| Fe_3_O_4_-10SiO_2_/Pt(300) | 0.93 | 10 | 1:11.25 | 1.4 | 300 | 1.78 |
| Fe_3_O_4_-15SiO_2_/Pt(300) | 0.93 | 15 | 1:16.92 | 1.4 | 300 | 1.80 |
| Fe_3_O_4_-20SiO_2_/Pt(300) | 0.93 | 20 | 1:22.50 | 1.4 | 300 | 1.68 |
| Fe_3_O_4_-25SiO_2_/Pt(300) | 0.93 | 20 | 1:28.29 | 1.4 | 300 | 1.79 |

**Table S2.** Performance details for benchmark experiments

| Catalyst | Catalyst loading [C:U]/ [g/g] | Amount of U (VI)/M | H_2_ pressure/bar | Time taken for near complete reduction/min | References |
| --- | --- | --- | --- | --- | --- |
| PtO_2_ | 1:10 | 0.42 | 33 | 45 | ^1^ |
| 2 % Pt/Al_2_O_3_ | 1:55 | 0.42 | 33 | 50 | ^2^ |
| 2 % Pt/SiO_2_ | 1:100 | 0.42 | 35 | 100 | ^2^ |
| 1 % Pt/SiO_2_ | 1:4000 (0.25 mg of Pt) | 0.42 | 2 | 40 | ^3^ |
| Fe_3_O_4_-10SiO_2_/Pt(200) | 1:4000 (0.25 mg of Pt) | 0.42 | 2 | < 35 | This Work |
| Fe_3_O_4_-10SiO_2_/Pt(250) | 1:4000 (0.25 mg of Pt) | 0.42 | 2 | < 30 | This Work |
| Fe_3_O_4_-10SiO_2_/Pt(300) | 1:4000 (0.25 mg of Pt) | 0.42 | 2 | ⁓ 30 | This Work |
| Fe_3_O_4_-15SiO_2_/Pt(300) | 1:4000 (0.25 mg of Pt) | 0.42 | 2 | < 35 | This Work |
| Fe_3_O_4_-20SiO_2_/Pt(300) | 1:4000 (0.25 mg of Pt) | 0.42 | 2 | < 35 | This Work |
| Fe_3_O_4_-10SiO_2_/Pt(300) | 1:4000 (0.25 mg of Pt) | 0.42 | 2 | < 40 | This Work |





**Figure S1.** XRD patterns of Fe_3_O_4_-10SiO_2_/Pt(150) (a), Fe_3_O_4_-10SiO_2_/Pt(200) (b), Fe_3_O_4_-10SiO_2_/Pt(250) (c) and Fe_3_O_4_-10SiO_2_/Pt(300) (d)





**Figure S2.** XRD patterns of Fe_3_O_4_-10SiO_2_ (a), Fe_3_O_4_-15SiO_2_ (b), Fe_3_O_4_-20SiO_2_ (c) and Fe_3_O_4_-25SiO_2_ (d)





**Figure S3.** XRD patterns of Fe_3_O_4_-10SiO_2_/Pt(300) (a), Fe_3_O_4_-15SiO_2_/Pt(300) (b), Fe_3_O_4_-20SiO_2_/Pt(300) (c) and Fe_3_O_4_-25SiO_2_/Pt(300) (d)


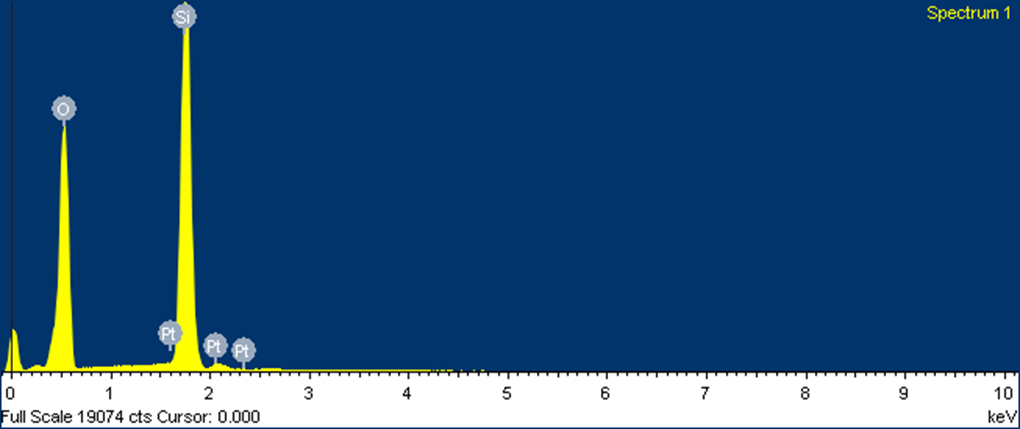


**Figure S4.** XEDS Spectra of Fe_3_O_4_-10SiO_2_/Pt(200)


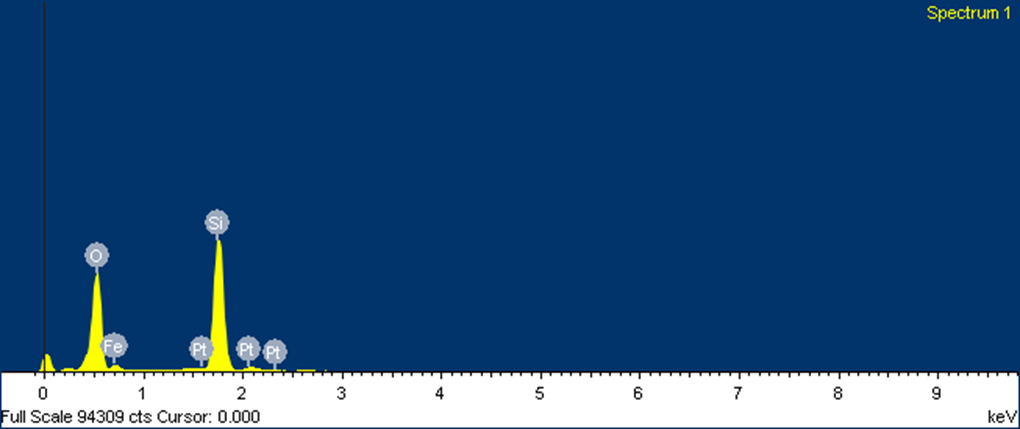


**Figure S5.** XEDS Spectra of Fe_3_O_4_-10SiO_2_/Pt(250)


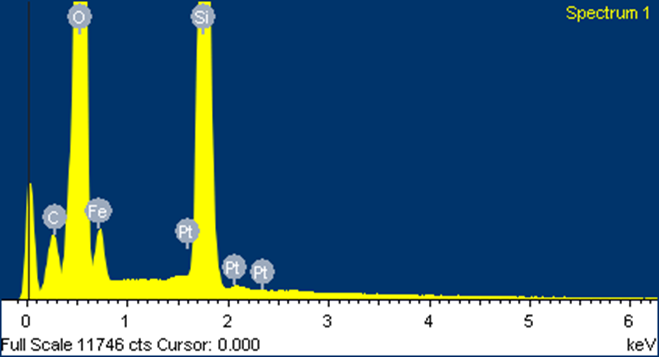


**Figure S6.** XEDS Spectra of Fe_3_O_4_-10SiO_2_/Pt(300)


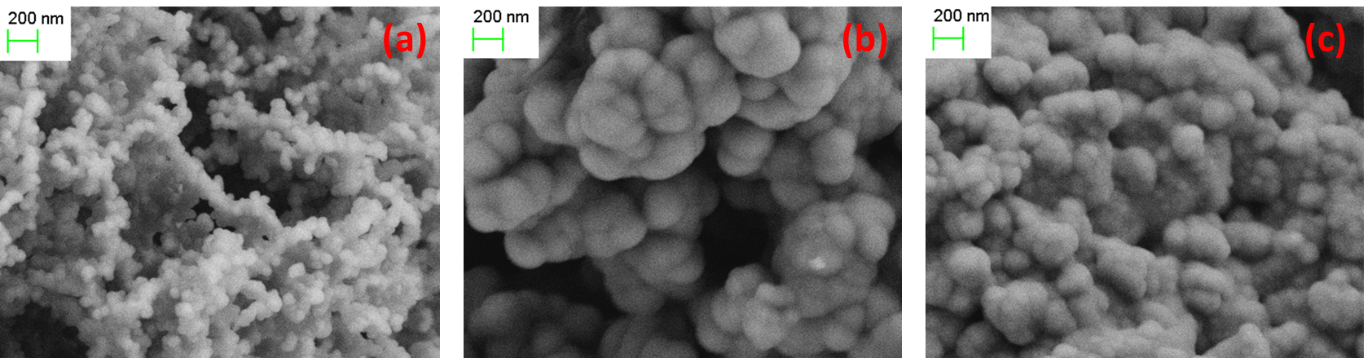


**Figure S7.** FESEM image of Fe_3_O_4_-15SiO_2_/Pt(300) (a), Fe_3_O_4_-20SiO_2_/Pt(300) (b) and Fe_3_O_4_-25SiO_2_/Pt(300) (c)


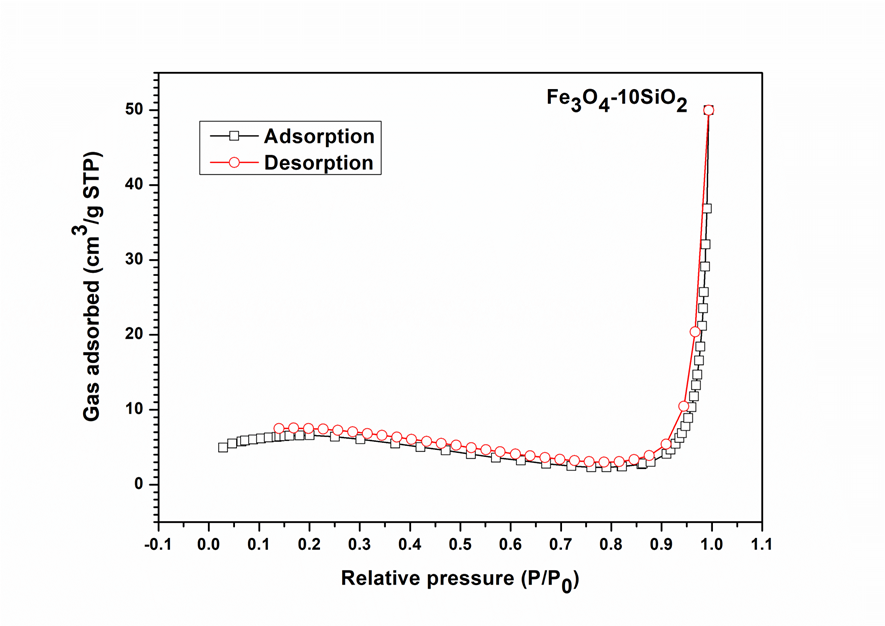


**Figure S8**. BET surface area of Fe_3_O_4_-10SiO_2_





**Figure S9.** BET surface area of Fe3O4-10SiO2/Pt


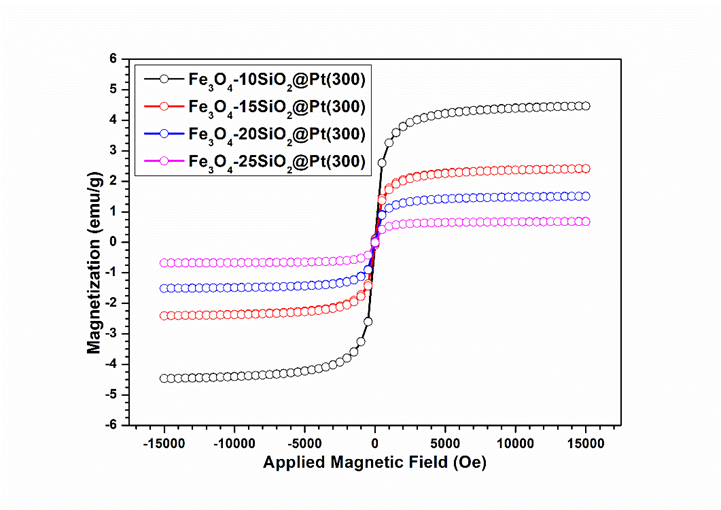


**Figure S10.** Variation in the magnetization with applied magnetic field of Fe_3_O_4_-10SiO_2_/Pt(300), Fe_3_O_4_-15SiO_2_/Pt(300), Fe_3_O_4_-20SiO_2_/Pt(300) and Fe_3_O_4_-25SiO_2_/Pt(300)





**Figure S11.** Plot of U(IV) conc. over time without any catalyst (Blank)





**Figure S12.** Plot of U(IV) conc. over time using Fe_3_O_4_-10SiO_2_ as catalyst





**Figure S13.** Plot of U(IV) conc. over time using Fe_3_O_4_-10SiO_2_/Pt(200) as catalyst





**Figure S14.** Plot of U(IV) conc. over time using Fe_3_O_4_-10SiO_2_/Pt(250) as catalyst





**Figure S15.** Plot of U(IV) conc. over time using Fe_3_O_4_-10SiO_2_/Pt(300) as catalyst





**Figure S16.** Plot of U(IV) conc. over time using Fe_3_O_4_-15SiO_2_/Pt(300) as catalyst





**Figure S17.** Plot of U(IV) conc. over time using Fe_3_O_4_-20SiO_2_/Pt(300) as catalyst





**Figure S18.** Plot of U(IV) conc. over time using Fe_3_O_4_-25SiO_2_/Pt(300) as catalyst


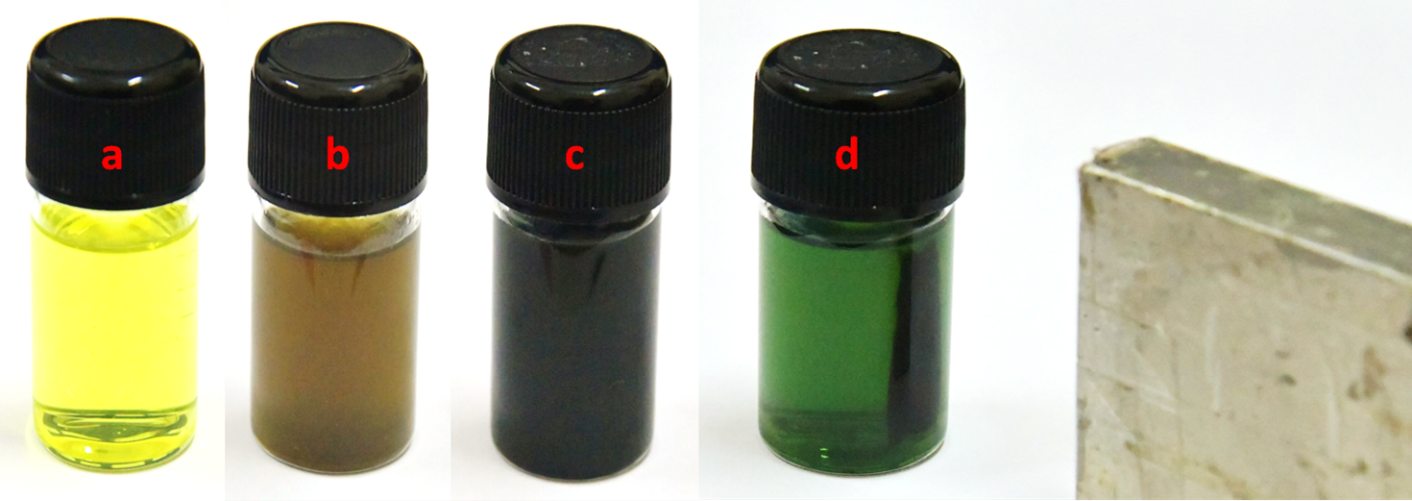


**Figure S19.** Digital Photograph of U(VI) stock solution 0.26 M (a), U(VI) Solution after addition of catalysts (b), U solution after completion of the reaction with catalyst dispersed (c) and U solution after separation of the catalyst with the help of a magnet (d)

**Reference:**

(1) Sahu, A.; Vincent, T.; Shah, J. G.; Wattal, P. K. Reduction of uranium (VI to IV) by hydrogenation using Adams’ catalyst. *Desalination and Water Treatment* **2014**, *52* (1-3), 426-431. DOI: <https://doi.org/10.1080/19443994.2013.808446>.

(2) Sahu, A.; Vincent, T.; Shah, J. G.; Wattal, P. K. Catalytic reduction of U(VI) to U(IV) using hydrogen with platinum loaded on alumina and silica. *Journal of Radioanalytical and Nuclear Chemistry* **2014**, *300*, 163–167.

(3) Reddy, S. R.; Ayyappa, S. V. N.; Mishra, S.; Sreepriya, T.; Desigan, N.; Venkatesan, K. A.; Sivaraman, N.; Ananthasivan, K. Effect of temperature and pressure on the reduction of U(VI) to U(IV) by hydrogen over Pt/SiO2: Reaction kinetics and modeling. *Chemical Engineering Research and Design* **2023**, *193*, 613-625. DOI: <https://doi.org/10.1016/j.cherd.2023.03.053>.
